# Observations on the Treatment of Lateral Curvature of the Spine, &c. &c.

**Published:** 1847-10-01

**Authors:** 


### Observations on the Treatment of Lateral Curvature of the Spine, pointing out the Advantages to be gained by placing the Body in a Position to produce Lateral Flexion of the Vertebral Column, combined with the after application of Firm Mechanical Support. With Woodcuts. By *E. F. Lonsdale*, F.R.C.S.E., &c. &c. 8vo. pp. 116. Churchill: London, 1847.

Every medical man knows that Lateral Curvature of the Spine is of much more frequent occurrence among the young females of our middle and upper classes than it ought to be; but we are certainly not prepared to believe, with our author, that " some deviation from the natural erect line, causing a greater fulness on the right than on the left side, both in the ribs and shoulder," exists " in by far the majority of them." It is unnecessary to allude to the usual predisposing and producing causes of this, unquestionably too common, deformity. The convexity of the curvature is, as just stated, very generally on the right side. The much greater use of the right arm, and indeed of the whole right half of the body, in most exercises, is probably the main cause of this. Mr. Lonsdale suggests that the greater expansion of the Lung on the right than on the left side may have also something to do with it. Speaking of the injurious effects of stays, he remarks:

" The constriction of the stays at first tells equally on both sides of the chest; but will it continue to do so after being allowed to act for a length of time and in an increasing degree? I say, no; for the simple reason that the ribs on the two sides do not offer the same resistance, owing to the difference in the size of the two lungs; the left side of the chest contains less air than the right, which it must, owing to the left lung being smaller than the right. Combined with this, there is the position of the Liver on the right side to be considered, to which I shall refer again, as a cause tending to support the right side of the chest, to render the ribs less liable to compression on this side than on the left. The constriction, then, produced by the force of the stays, must act more on the side where there is the less resistance, and this, as already stated, is the left; the consequence of which is, that the ribs of this side will become more compressed, and the capacity of the lung be also diminished." P. 11.

The supposed influence of the Liver existing on the right side is thus more largely explained:

"I would ask the question, may not the liver, from its position, give a degree of support to the under surface of the ribs of the right side, sufficient to cause a difference in the resistance of the two sides of the thorax? Does it not mechanically, to a certain extent, prevent the ribs from being depressed, while on the left side no such support or resistance exists? Let any one stand in the erect position, and try to flex the spine laterally, he will find he can do so with much greater facility and to a far greater extent on the left than on the right side. May not this be owing to the presence of the liver on the right, and to its absence on the left side? It may be said, that the liver will be depressed, and so the opposition removed. I believe it will not; for any movement of the spine which bends it to one side or to the other, is principally produced by the action of the abdominal muscles; they necessarily press against the viscera at the same time, and so oppose the displacement of the liver; and not only oppose it, but in extreme action tend to press it upwards. If so, it is quite intelligible how the liver must give support to the under surface of the ribs, with which it is in contact." P. 20.

The description of the symptoms, the origin and progress, of the deformity is minutely and faithfully given by Mr. Lonsdale, and may be read with much advantage by those whose attention has not hitherto been directed to the subject.

With respect to the principles of Treatment recommended by our author, the reader will be best enabled to judge of them from the following remarks:

"First,—there is weakness of the vertebral column; it has then to be artificially supported. Second,—there is displacement of certain bones; the vertebræ, the ribs, scapulæ, and clavicles, which lose their natural relative position to one another, at the same time that the ligaments on one side become shortened; these bones then have to be replaced, and the resistance of the ligaments has to be overcome. Finally, there is irregular muscular development of the two sides of the body, existing both as a cause and as an effect. These points then have to be considered; and the plan of treatment which will most effectually gain the desired end, is the one to be pursued. My own conviction is, that they must, in most cases, all three be combined; that no one of them will be effectual if employed alone; that is to say, it is no use supporting the spine without the displaced bones are mechanically acted on with the intention of replacing them, at the same time that means are taken to overcome the resistance of the ligaments; and that it is no use doing either of these, without the action of the muscles be attended to afterwards, by endeavouring to give increased power where it is deficient. On the other hand, it is of little use attending to the muscular system only, which is done in many plans of treatment; for it by itself is not sufficient to redress the deformity, but in many cases, as stated before, will only tend to increase it, if the spine be not first of all brought out of its curved position; a point which can be easily understood if the origin and the insertion of the muscles are considered, and the action they will have upon the ribs and spine, when these bones are thrown so much out of their relative position. I shall first explain the means by which the spine can be well and efficiently supported: afterwards, the means to be adopted by which the resistance of the ligaments can be most effectually overcome, at the same time that the bones are pressed in a direction the opposite to that in which they are displaced: finally, the advantages that are to be gained by the exercise of the muscles, and the position best adapted to increase their development, as well as to act upon the spine itself." P. 55.

The spinal apparatus or support used by Mr. Lonsdale is a modification of that recommended by Mr. Tamplin in his work on Deformities, and certainly seems to be exceedingly well suited to its purpose; that of supporting the left or depressed shoulder, and of exercising a degree of steady pressure upon the expanded and projecting ribs on the right side, and, through them, upon the convexity of the spine itself. Its construction and mode of acting are well represented in several woodcuts.

While disapproving of the use of any modification of the recumbent position, when continued for months and years, as has been so often recommended and adopted—Mr. Lonsdale adopts it as an adjunct only in the treatment of lateral curvature. But, in the employment of this means, he does not follow the ordinary plan of placing the patient either on the back or on the stomach; in other words, either in the supine or in the prone attitude. He thinks that the position to be adopted may be made a powerful means of redressing the bend of the spinal column, and he reasons in this way. If you wish to straighten a curved stick do you stretch it by pulling upon the two ends in its long axis?—no; you place the convex of the bend upon your knee and then pull or draw back the two ends. The same principle may be carried out in the case of the curved spine, says our author; and in this way:

"The patient should be placed on the side, on which the projection formed by the curve exists, instead of on the back, and allow the legs, the head, and upper extremities, to fall to a lower level than the trunk; by this means a sufficient power is at once gained, by the simple weight that is then exerted at either end of the trunk, to gradually act upon the spine and to regulate itself; imitating, in fact, the straightening of a bent rod or stick: no other mechanical means are required; the weight of the legs at the one extremity, and of the head and shoulders at the other, exert a force quite sufficient to redress any slight curvature that may exist, and as much as can be borne, or it may be judicious to apply in severer cases. The object is to stretch the ligaments, and so to overcome their resistance, at the same time that the bones themselves are pressed in a direction the opposite to that in which they have been displaced, and are thus rendered more moveable and more capable of being acted upon, by any apparatus that may be afterwards employed to give them support,—points to be more particularly referred to, and which I explain after describing the means by which this position is to be obtained." P. 84.

The couch contrived by our author cannot be properly explained without the aid of his diagram. It is ingenious, and must answer the purpose for which it was planned very well; but we are much disposed to think that the simple plan used by Mr. Alexander Shaw—that of placing a firm pillow or cushion beneath the projecting side of the thorax, the patient reclining upon this side and the pelvis and lower extremities resting on a somewhat inclined plane—must effect nearly all the advantages to be derived from the position recommended. "The simple object is to render the spine more flexible; to make it more yielding in the opposite direction to that in which it is curved; to overcome the resistance of the ligaments by gradually stretching them; to render the bones themselves more moveable, by pressing against them in the direction the opposite to that in which they have been displaced,—both the vertebræ themselves as well as the ribs; and at the same time to overcome the resistance of the muscles which have hitherto been acting in one direction only,—imitating, in fact, the treatment of the muscles and ligaments in a contracted joint."

To gain these ends, an hour or two's reclination daily in the position recommended is all that is necessary. On the patient's rising, the instrument already described must at once be put on, "to *retain any advantage that may have been gained*, and to support the spine at once after having been thus bent in the opposite direction, making the instrument act more and more every day." So much for the two mechanical means, recommended by our author for *unbending* the curve of the spine, and retaining it in its normal uprightness. Let us now hear what he says respecting the third and last set of means which he describes, that of the exercise of the muscles. At page 80, he expresses himself as anything but favourable to this part of the treatment in a very great number of cases.

"I am opposed to the exercise of the muscles before the curvature has been either completely cured, or as much relieved as the nature of the case will admit of, for the reasons already adduced when speaking of the causes of lateral curvature, namely, that the muscles themselves, after the curvature is once produced, tend by their action to increase it; and I more particularly refer to the spinal muscles, both those which are proper to the spine itself, and those which are connected with the ribs; they both draw the concave side of the spine more and more downwards, the more powerful the action they exert. For this reason, then, I should avoid increasing their power so long as any curvature may exist and the spine itself be yielding, and therefore should not combine it with the stretching position, did I think that one the best adapted to mechanically overcome the deformity. This view appears to me to be rational, and I am not aware it has been before advanced, except by Mr. Tamplin, to whose lectures I have before referred." P. 80.

And a few pages further on, he remarks—"that to exercise the muscles of the back, including those of the upper extremities, without mechanically relieving the spine of its superincumbent weight, does more harm than good, and that it increases the deformity rather than relieves it; and that what is termed general increase of muscular development, by indiscriminately employing gymnastic exercises, is not to be recommended. I now more particularly refer to those cases of lateral curvature, where the deformity does not depend on weakness of the muscles as the principal cause, but upon confirmed and mechanical displacement of the bones of the vertebral column and ribs."

While admitting that a few cases of lateral curvature of the spine may be cured by "attention to muscular exercise—properly employed," (including, we presume, frequent rest in the horizontal posture), Mr. Lonsdale affirms that, in the majority of cases, this plan of treatment will fail: "the muscles of the weak side may have been increased in power, but the deformity may not have been relieved." The only cases—and they are declared to be *few* in point of number—in which muscular exercise is of much service, are said to be those occurring "in girls of spare habit, in whom the whole muscular system is weak; where the vertebræ are thinly covered with the muscles, the spinous process being prominent throughout the whole length of the spinal column; the scapulæ on both sides projecting and wanting their close adaptation to the ribs, owing to the absence of sufficient power in the muscles to keep them in their natural position: where the curvature of the spine is general throughout its whole length, and can be easily altered in one direction or the other, the bones being but loosely connected owing to deficiency of strength in the ligaments; the shoulder of one side being higher than the other, though not to any marked extent, and the ribs of the left side, though less convex than on the right, still are not compressed to an extent sufficient to cause a hollow beneath the left scapula: lastly, where the curvature has existed for a short time only, and will admit of being easily redressed by pressure made with the hands."

Now compare such cases with another set, in which the exercise of the muscles is declared by our author to do more harm than good; viz.

> "Those where the curvature, although it may be confirmed, may not yet be fixed; by which I mean, the deformity may be very great, but yet there may be sufficient yielding in the spine to allow of it being moved or acted upon when pressure is made forcibly against it; where the ribs are more increased in convexity on the right side and more depressed on the left, with a corresponding projection and sinking of the scapulæ of the two sides, causing also the corresponding difference between the level of the two shoulders. *Any increased power given to the muscles in these cases, without attempting mechanically to support the ribs and spine, and to support the left shoulder,* which by its weight is tending to bear downwards and to increase the concavity, *will,* as before stated, *only keep up the deformity, and in the majority of cases increase it.* This opinion, I am aware, is at variance with that which is advocated by those who look to increased muscular development as an important point in the treatment of these cases; it is formed upon the reasons I have already given, and from my own experience I believe it to be a correct one. The grand point to attend to, is to bring the spine as nearly as possible into its normal erect line; to relieve the compressed ribs of the left side, by supporting the shoulder which is bearing upon them with its weight, at the same time that the opposite or convex side is pressed upon by a force that gradually admits of being increased." P. 102.

It will be observed, from the passage we have had printed in Italics, that our author somewhat qualifies his opinion as to the hurtful effects of exercises even in these cases. As a matter of course, when the deformed vertebral column has become, from the long-standing of the case and the age of the patient, rigidly fixed in its altered position, no rational man can expect that any sort of muscular exercise can ever correct the evil. All that can then be done is to have recourse to a well-devised mechanical support, frequent reclination in the horizontal position, and great attention to the general health.

The exercise, we should have remarked, recommended by our author, will be best understood from the following passages:—

> "The position that I believe to be the best, is one that throws the whole spine more backwards than forwards; which tends to redress the curvature, at the same time that the muscles of the spine are brought actively into play; and the following is the one I should recommend. Attach two pullies or hooks (and pullies answer the purpose better) to the ceiling of the room, or to an artificial frame-work placed in some situation about two or three feet above the head. The patient is to stand in a position, that the pullies may be about a foot and a half or two feet behind her. She is then with both hands to take hold of a stick or spindle, to which two ropes are attached, and which pass through the pullies, having weights fastened at the other ends sufficiently heavy to require some exertion to draw them up, the weights of course being increased or diminished according to the strength of the patient. I generally find six or eight pounds in each quite enough, and as much as the patient can raise without over-fatiguing herself. The ropes should be long enough to allow her to incline the body forwards on the hip-joints, without bending the spine itself, drawing the weights upwards as much as she can, keeping the arms extended above the head all the time, and bringing them as far forwards as the inclination of the body will admit of, without the necessity of moving the feet from the position in which they were originally placed. The body is then to be brought into the erect position again, by raising the trunk on the hip-joints, and letting the weights fall, and so to pull the arms behind and above the head. It may be as well to tie a knot in the ropes, to check the fall of the weights, to prevent the arms being strained beyond the point of extension to which they can easily be carried behind the head." P. 108.

This exercise should often be taken with the left arm only. He adds:

" The grand principle I wish to lay down, is, to exercise the muscles with the arms placed above and behind the head, while the body is kept in the erect, and not in the horizontal position. If this principle be well carried out, and a strong and efficient spinal support be employed at the same time, I believe that all *slight* cases of lateral curvature may be cured without the necessity of employing couches at all." P. 112.

In taking our leave of Mr. Lonsdale's work, we cannot but express our opinion that he seems disposed to attach a somewhat exaggerated importance to the use of mechanical means, and to undervalue the benefits to be derived from the judicious employment of muscular exercises, in the treatment of lateral deformities of the spine. With this qualification, we think that many of his suggestions are sound, and well deserving of the attention of the profession.

